# Association of the inflammatory marker suPAR with chronic pruritus of unknown origin – data from the SOMA.PRU study

**DOI:** 10.3389/fimmu.2026.1804748

**Published:** 2026-06-16

**Authors:** Stefan M. Kahnert, Lara Jürgens, Leonie Dreher, Christian Schmidt-Lauber, Ansgar Koechel, Gina L. Frank, Tobias B. Huber, Meike Shedden-Mora, Sonja Ständer, Markus Ramm, Gudrun Schneider, Rupert Conrad

**Affiliations:** 1 Department of Psychosomatic Medicine and Psychotherapy, University Hospital Münster, Münster, Germany; 2 III. Department of Medicine, University Medical Center Hamburg-Eppendorf, Hamburg, Germany; 3 Hamburg Center for Kidney Health (HCKH), University Medical Center Hamburg-Eppendorf, Hamburg, Germany; 4 Institute for Clinical Psychology and Psychotherapy & Department Psychology, Medical School Hamburg (MSH), Hamburg, Germany; 5 Department of Psychosomatic Medicine and Psychotherapy, Centre for Internal Medicine, University Medical Centre Hamburg-Eppendorf, Hamburg, Germany; 6 Department of Dermatology, Section Pruritus Medicine and Center for Chronic Pruritus (KCP), University Hospital Münster, Münster, Germany

**Keywords:** chronic inflammation, chronic pruritus, persistent somatic symptom, soluble urokinase plasminogen activator receptor, suPAR

## Abstract

**Background:**

Chronic pruritus (CP) is a frequent and burdensome dermatological symptom that can occur both in skin conditions such as chronic atopic dermatitis (cAD) and as CP on non-lesional skin of unknown origin (CPUO). Mechanisms of pruritus chronicity remain poorly understood. In line with other persistent somatic symptoms, chronic systemic inflammation may play a role in CP. Soluble urokinase plasminogen activator receptor (suPAR) has been proposed as a marker of long-term systemic inflammation, but has not yet been studied in relation to CP.

**Objective:**

To study serum levels of suPAR in patients with CPUO in comparison to cAD and to study possible associations between suPAR levels and prospective clinical course.

**Methods:**

We analyzed data from 105 participants (35 CPUO, 40 cAD, 30 controls). Clinical and patient-reported outcomes were collected at baseline and at a six-months follow-up visit.

**Results:**

Patients with CPUO had the highest suPAR levels in our study (3.2 ± 2.5 ng/ml), showing a non-significant trend toward higher levels than controls (2.9 ± 1.1 ng/ml) and, despite similar pruritus intensity, significantly higher than participants with cAD (2.0 ± 0.9 ng/ml, *p* = 0.018). Additionally, CPUO participants with higher levels of suPAR at baseline had higher average and worst pruritus intensity in the follow-up visit.

**Conclusion:**

This study indicates that systemic chronic inflammation is a relevant aspect of CPUO. In particular, we infer that suPAR may be a biomarker associated with the longitudinal course of CPUO.

## 1 Introduction

Chronic pruritus (CP, lasting ≥ 6 weeks) is a frequent dermatological symptom that affects 15% to 20% of the general population (1, 2) and greatly reduces quality of life (3). CP can occur both in well-defined skin conditions such as atopic dermatitis (AD) and as chronic pruritus on non-lesional skin without clear attribution to an underlying disease or pathomechanism (chronic pruritus of unknown origin; CPUO) (4). CPUO affects about 6-15% of pruritus patients and rises with age (5).

CP can be understood as a prototypical example of a persistent somatic symptom (PSS), describing subjective, distressing, chronic somatic complaints that affect quality of life (6). Overall, mechanisms of chronicity both of PSS in general and pruritus in the patient groups described above are incompletely understood (7, 8), but include biological, psychological and social aspects (6, 9).

Of these, systemic chronic inflammation is of particular interest. Chronic systemic inflammation is increasingly recognized as a state of persistent, low-grade immune activation that may arise from both age-related processes and the cumulative burden of comorbidities. In patients with CP, systemic inflammatory signatures may therefore reflect not only cutaneous immune activation but also underlying comorbidity profiles. However, the extent to which localized or widespread skin inflammation translates into measurable systemic inflammation remains incompletely understood and likely depends on multiple factors, including disease severity, duration, and individual susceptibility.

Systemic chronic inflammation has not only been linked to PSS (7, 10), but might also be the mechanism linking psychosocial risk factors and somatic symptoms: E. g., traumatic/adverse life events, particularly early in life, have repeatedly been associated with PSS (10–12) and also with chronic inflammation (13) and several other conditions that come along with chronic inflammation (14).

Some behavioral aspects such as obesity or smoking have been linked to PSS (9) and also contribute to systemic chronic inflammation (15). In addition to dermatoses such as chronic nodular prurigo (16), CP in systemic entities such as chronic kidney disease (CKD) has also recently been linked to the presence of systemic inflammation (17). CPUO has also been linked to Th2 inflammation based on skin investigations and to altered blood amino acid metabolites (18, 19). However, to date, no study investigated a validated biomarker for systemic chronic inflammation in CPUO.

Since chronic inflammation is a major driver for development, progression and outcome of a wide variety of diseases, its determination is highly relevant for understanding, characterization and treatment of individual disease stages (20). In the past, the main biomarker to assess inflammation was serum C-reactive protein (CRP), but its utility as a marker of chronic inflammation is limited because its levels rise quickly and markedly in response to acute infections (21). Additionally, CRP can also be influenced by recent physical exertion (22), mental stress or some medications (23).

Meanwhile, another immune mediator, the soluble urokinase plasminogen activator receptor (suPAR) has recently been described as a more stable biomarker for systemic chronic inflammation in various diseases (24–26), as it is less influenced by acute changes than CRP (21). So far, suPAR has predominantly been investigated in somatic illness such as CKD and for objective health outcomes like mortality (26, 27), whereas its role in subjective complaints and PSS has scarcely been studied. A recent study found higher levels of suPAR in a small cohort of patients with chronic pain (28), but associations between suPAR levels and the severity, progression or outcome of pruritus have not been investigated.

Our study aims to investigate suPAR as a marker of chronic inflammation and disease intensity and outcome in patients suffering from CP on skin with (AD) and without (CPUO) lesions. We hypothesized that suPAR levels would be higher in patients with chronic AD (cAD) and CPUO than in HC and that in the patient cohorts, those with higher levels of suPAR would have higher pruritus intensity, both cross-sectionally and after a follow-up of six months.

## 2 Materials and methods

The research presented here is an adjunction to the longitudinal, observational SOMA.PRU study, which aims more broadly at identifying biopsychosocial risk factors of pruritus persistence, see 8 for the published study protocol.

### 2.1 Participants

Participants with cAD and CPUO were recruited at a university dermatology and pruritus outpatient department in Münster, Germany as part of the SOMA.PRU study. Common inclusion criteria were age ≥ 18 years, sufficient German language skills and for cAD and CPUO worst pruritus intensity ≥ 5 on a numeric rating scale (NRS, range 0–10) in the last 24 hours. cAD participants were required to have chronic eczema for at least six weeks, whereas CPUO participants were required to have no clinical phenotype such as dermatosis or chronic prurigo. Common exclusion criteria included florid psychosis, acute suicidality, pregnancy/breast-feeding and intake of systemic steroids or immunosuppression in the last 4 weeks. HC were required not to have any skin conditions, pruritus in the last 24 hours or atopic diathesis. All inclusion and exclusion criteria were checked by a dermatologist. Due to the integration of this project into the larger SOMA.PRU study, no formal *a priori* sample size calculation was performed for this study. The SOMA.PRU study also recruited participants with acute AD, but they were not investigated for this project, as they do not necessarily suffer from CP or PSS. All participants provided written informed consent for the overall SOMA.PRU study before enrollment and an Ethics Amendment for the additional analysis of the biomarkers presented here was accepted by the independent review board (Ethics Committee of the Medical Association of Westphalia-Lippe, Münster, Germany; No. 2020-676-f-S).

### 2.2 Patient-reported outcomes

Participants answered a set of self-report questionnaires in German, usually on a tablet computer programmed in REDCap. The data collected for this analysis included basic sociodemographic information as well as average and worst pruritus intensity in the last 24 hours on the NRS, ranging from 0 ‘no pruritus’ to 10 ‘worst pruritus’. Participants were invited for a follow-up visit after six months (6MFU), when most assessments were repeated.

### 2.3 Biological parameters

All participants were assessed by a dermatologist to confirm attribution to cAD, CPUO and HC, who also calculated the Scratch Sign Score (SSS) based on type and extent of scratch lesions (affected body surface area, BSA) as described previously (29).

Blood was drawn from all participants at the baseline (BL) visit, centrifugated and serum was stored at -80 °C before analysis. suPAR levels in serum were measured using the suPARnostic Elisa Kit (ViroGates A/S, Denmark) following the manufacturer’s guidelines. Based on quality standards, all measurements were performed in duplicate. Because of the known role of suPAR in CKD, serum creatinine was also measured (ultraviolet-visible spectrophotometry on the cobas c702 platform, Roche, Germany) and subjects were excluded if creatinine levels were above the upper limit of normal provided by the laboratory (age- and sex-based cut-offs: women < 1.1 mg/dl, men ≤ 50 years < 1.3 mg/dl, older men < 1.4 mg/dl).

### 2.4 Statistical analyses

For statistical analysis, unpaired *t*-tests were used for comparison of two quantitative variables and an analysis of variance (ANOVA) with a *post-hoc* test was used for comparison of three quantitative variables, both provided that normal distribution could be assumed. Effect sizes are reported as Cohen’s *d*. Fisher’s exact test was used for categorical, sociodemographic variables due to low expected cell counts. Analyses of covariance (ANCOVA) were calculated to examine possible confounders (including age groups: < 65 years vs ≥ 65 years) and linear regression models were calculated to study predictive effects of quantitative variables. Correlations between quantitative variables were reported using Pearson’s *r*. Statistical analyses were performed using SPSS 28.0 (IBM SPSS Statistics for Windows, Version 28.0. Armonk, NY: IBM Corp). *p*-values < 0.05 were considered statistically significant.

## 3 Results

In total, 110 participants were recruited, five of which showed creatinine values over the normal upper limit (s. Section 2.3.) and hence might exhibit CKD-associated pruritus. Subsequently, these participants were excluded from further analysis, leading to a final sample size of *n* = 105 (40 cAD, 35 CPUO, 30 HC). The three study groups were comparable in sex distribution and smoking behavior. However, patients with cAD were younger and had a lower BMI compared to CPUO patients.

**Figure 1:**
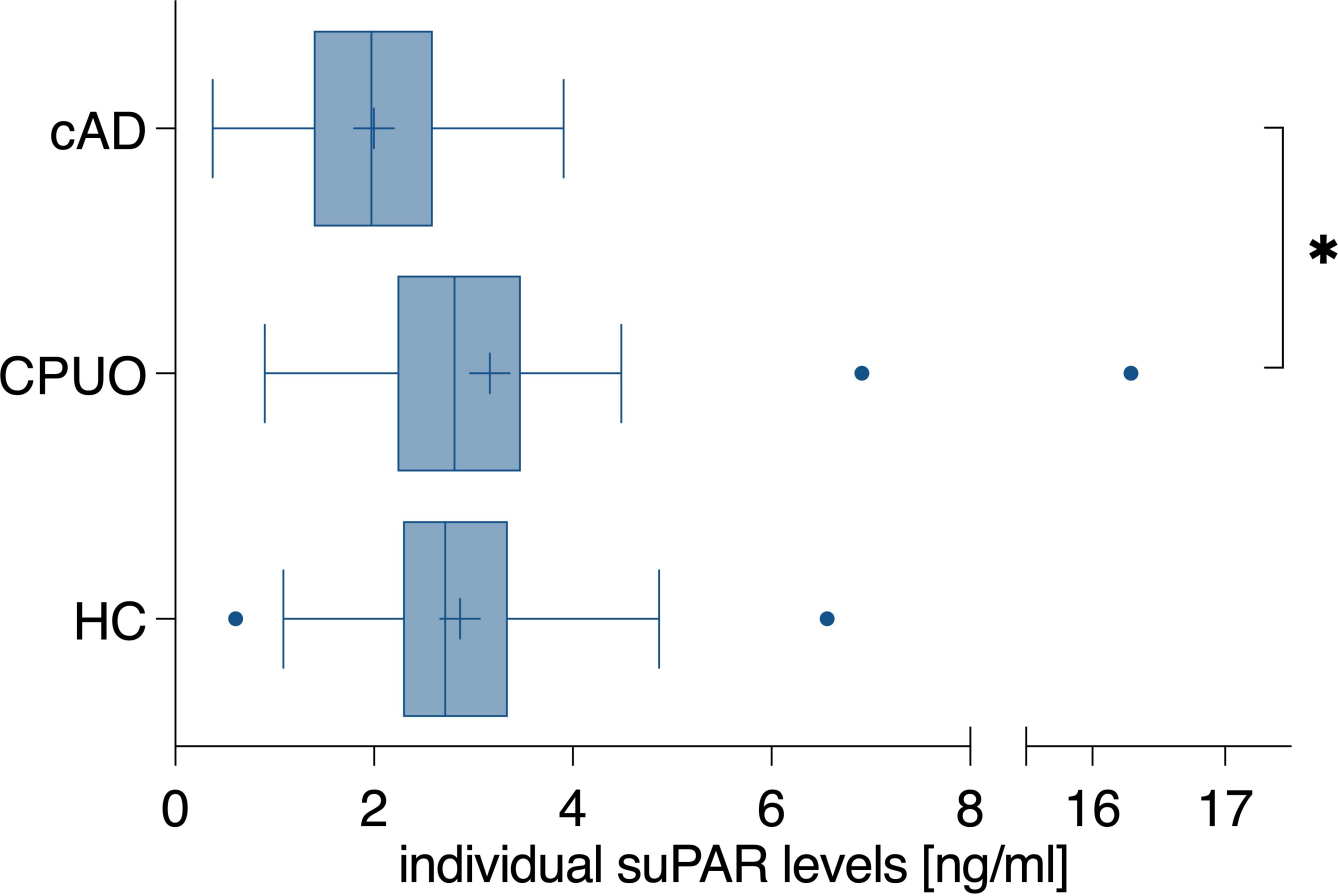
Serum levels of soluble urokinase plasminogen activator receptor (suPAR) in patients with chronic atopic dermatitis (cAD) and chronic pruritus of unknown origin (CPUO) as well as healthy controls (HC). Tukey box plots represent the upper and lower quartiles and the median. Mean is represented by the plus sign. *p < 0.05.

Measuring suPAR concentrations, the highest level of this nonspecific inflammation marker was detected in patients with CPUO, compared to cAD and healthy controls (**Figure 1**). An ANOVA revealed a significant difference between the three groups (F(2, 102) = 4.906, *p* = 0.011). *Post-hoc* tests (Bonferroni corrected for multiple comparisons) confirmed that the CPUO group showed significantly higher average suPAR levels than the cAD group (3.2 ± 2.5 ng/ml vs. 2.0 ± 0.9 ng/ml, *p* = 0.018, *d* = 0.66; Mdiff = 1.2; SE = 0.44). The other differences were non-significant; suPAR levels of HC were 2.9 ± 1.1 ng/ml. Comparing patients with CPUO and cAD, both groups showed similar levels of mean average and worst pruritus intensity and did not differ significantly in terms of sex distribution, smoking behavior or creatinine (**Table 1**). Participants with cAD had significantly higher SSS values. As a component of this, they also exhibited a larger affected BSA, with a mean affected BSA of 10-29% (data not shown).

**Table 1:** Sociodemographic and clinical data of 105 participants with chronic atopic dermatitis, chronic pruritus of unknown origin and skin-healthy controls.

Features	cAD		CPUO		HC		Significance (cAD vs. CPUO)
	n	M ± SD	n	M ± SD	n	M ± SD	
age	40	35.4 ± 19.4	35	64.0 ± 10.6	30	34.9 ± 13.6	*p* < 0.001
sex	40	26 female, 14 male	35	20 female 15 male	30	20 female 10 male	*p* = 0.64
smoking	34	no: 25 occasionally: 7 regularly: 2	30	no: 27 occasionally: 1 regularly: 2	29	no: 21 occasionally: 3 regularly: 5	*p* = 0.13
BMI	39	24.3 ± 3.7	34	28.0 ± 5.3	29	25.2 ± 4.9	*p* = 0.001
average pruritus	40	5.4 ± 1.7	35	6.0 ± 1.9	30	n. a.	*p* = 0.2
worst pruritus	40	7.1 ± 1.6	35	7.2 ± 1.9	30	n. a.	*p* = 0.9
SSS	39	3.0 ± 2.0	35	1.1 ± 1.7	30	n. a.	*p* < 0.001
suPAR	40	2.0 ± 0.9	35	3.2 ± 2.5	30	2.9 ± 1.1	*p* = 0.018
creatinine	40	0.8 ± 0.2	35	0.8 ± 0.2	30	0.8 ± 0.1	*p* = 0.05

BMI, Body Mass Index; cAD, chronic atopic dermatitis; CPUO, chronic pruritus of unknown origin; HC, skin-healthy controls; SSS, Scratch Sign Score; suPAR, soluble urokinase plasminogen activator receptor.

**Figure 2:**
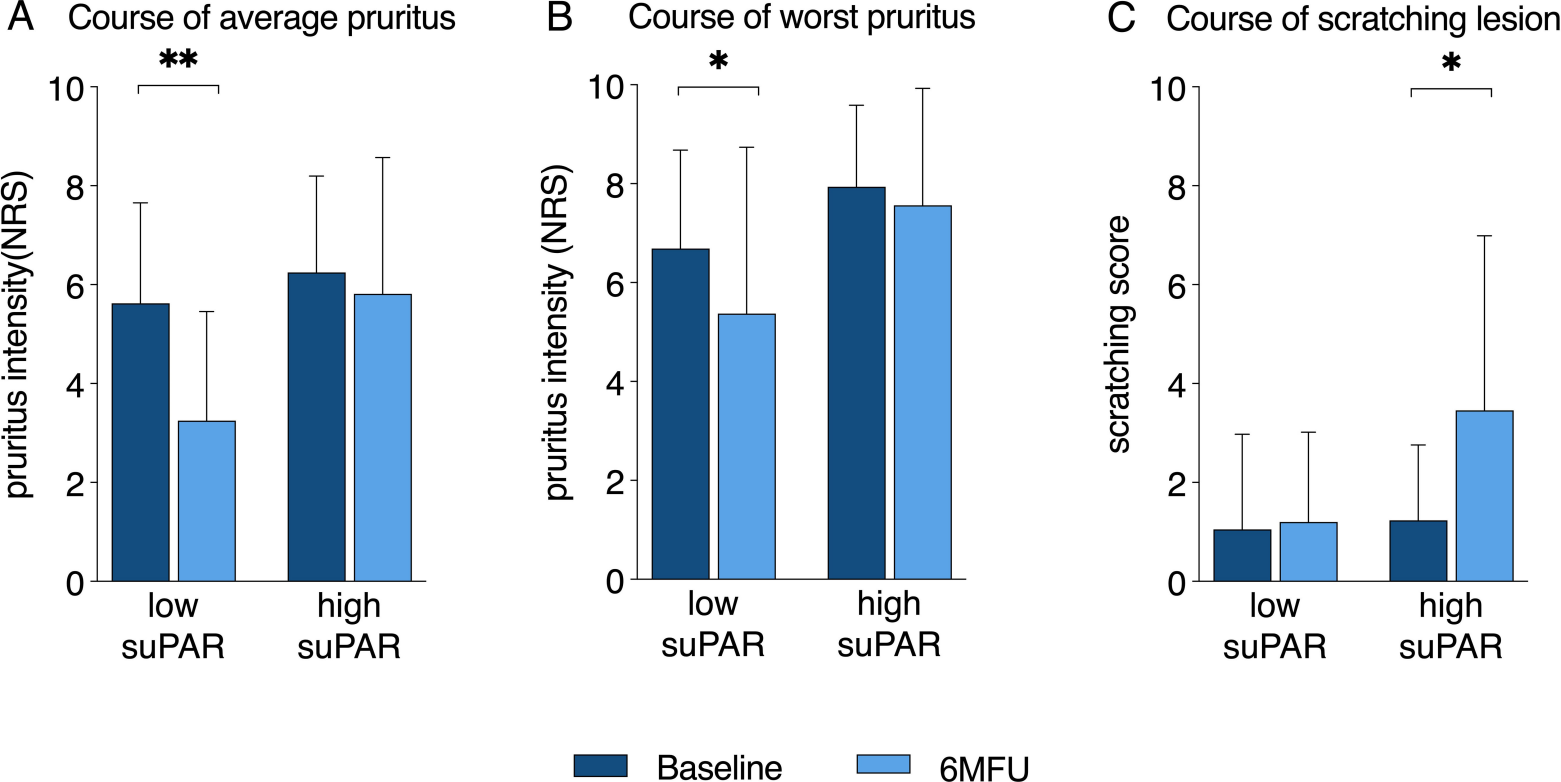
Intensity of **(A)** average and **(B)** worst pruritus as well as scratch lesions as determined by scratch sign score **(C)** both at baseline and 6-month follow-up (6MFU) in patients with chronic pruritus of unknown origin. Patients were subdivided in low and high suPAR level groups, based on a median split by measured suPAR levels (2.8 ng/ml). Pruritus intensity in the last 24 hours was measured by a numeric rating scale (NRS, scale 0-10). suPAR: soluble urokinase plasminogen activator receptor. *p < 0.05, **p < 0.01.

To evaluate the potential use of suPAR as a prognostic biomarker for the longitudinal course of CP, data from the 6MFU, which were available for 62 patients (33 cAD, 29 CPUO), were analyzed (**Supplementary Table 1**). Therefore, median splits of cAD (at 1.975 ng/ml) and CPUO (at 2.81 ng/ml) were performed dividing each group in ‘low suPAR’ and ‘high suPAR’ (**Supplementary Table 2**). The cAD groups did not differ significantly with regards to pruritus neither at baseline nor at the 6MFU. For CPUO, there was a trend to higher pruritus intensity in the high suPAR group at baseline, but this was not significant. However, the high suPAR group had higher average (5.6 ± 2.5 vs. 3.2 ± 2.4, *p* = 0.017, *d* = 0.96) and worst pruritus (7.3 ± 2.4 vs. 4.9 ± 3.4, *p* = 0.043, *d* = 0.81) at follow-up (**Figure 2**). Additionally, an increase of the SSS was detected between baseline and the 6MFU in high suPAR patients with CPUO. In the low suPAR group, a similar SSS was found (**Supplementary Table 2**).

In CPUO, after exclusion of two outliers with extreme suPAR values, linear regression analysis revealed a strong association between baseline biomarkers, baseline pruritus intensity and average pruritus at 6MFU. The model was highly significant (F(4,21) = 8.67, *p* < 0.001, adjusted R² = 0.55). Higher baseline suPAR (β = 0.55, *p* = 0.004) and creatinine (β = 0.34, *p* = 0.04), as well as younger age (β = –0.34, *p* = 0.04), were significant predictors of greater average pruritus (**Table 2**). The model predicting worst pruritus intensity at the 6MFU was not quite significant (F(4,21) = 2.25, *p* = 0.09). In cAD, none of the regression models including age, creatinine, and suPAR as predictors of average or worst pruritus reached statistical significance.

**Table 2:** Regression model for predictors of average pruritus intensity at 6-month follow-up in CPUO.

Dependent variable	Model	Baseline predictor	β	P-value
Average pruritus (NRS 24h, 6MFU)	F(4,21)=8.67, adj. R² = 0.55, p < 0.001	suPAR (ng/ml)	0.55	0.004
		Creatinine (mg/dl)	0.34	0.041
		Age (years)	-0.34	0.041
		Baseline pruritus (NRS 24h)	–	0.82

6MFU, 6-month follow-up; CPUO, chronic pruritus of unknown origin; NRS, Numeric Rating Scale; suPAR, soluble urokinase plasminogen activator receptor.

## 4 Discussion

This study focused on the evaluation of the new biomarker suPAR as a non-specific marker of chronic systemic inflammation in chronic pruritus. Therefore, patients suffering from cAD and CPUO were compared to skin-healthy controls for their blood suPAR levels at baseline, average and worst pruritus intensity and scratching scores.

We detected lower levels of suPAR in the cAD group compared to HC. Although this finding sounds unexpected, it is in line with the current literature (30). Additionally, the cAD cohort in our study showed low levels of acutely inflamed skin lesions. Peripheral blood samples of mild AD also showed no abnormalities in another study (31). In other inflammatory skin conditions, like psoriasis, suPAR was also not elevated (32, 33) further supporting the notion that these diseases are spatially confined rather than systemic in nature. In contrast to cAD, elevated suPAR levels were detected in patients with CPUO suggesting that CPUO may be better understood as a systemic inflammatory symptomatology rather than a disease limited to the skin. Interestingly, this was a marked, highly significant elevation when compared to cAD, despite very similar pruritus intensity. This might be of clinical and biological importance, as patients with CP on unchanged skin are similarly burdened as patients with cAD (34). Whereas some biomarkers are available for cAD (35), to our knowledge, biomarkers to describe the course of CPUO have previously not been available or were only described in small cohorts without validation. For example, in 11 patients with CPUO, a blood mass-spectrometry analysis revealed several elevated amino acid metabolites related to catecholamine biosynthesis and tryptophan biosynthesis (19). The significance of these findings is yet unknown.

Thus, suPAR might firstly be valuable for discriminating CPUO from other pruritic diseases including cAD. The relative insensitivity of suPAR to acute inflammation as compared to e. g. CRP (21) increases its clinical utility, meaning that patients with CP can be assessed despite e. g. mild respiratory tract infections or single inflamed scratch lesions.

Preliminary research has linked CPUO to inflammatory processes. The presence of Th2 inflammation in the skin of patients with CPUO has been described (18). Even in the absence of visible skin changes, low-grade systemic or localized immune activation may contribute to altered nerve signaling and skin sensitivity, leading to persistent itch (36). This line of thought is supported by our findings of elevated suPAR levels in CPUO, with suPAR being a validated biomarker for presence of general systemic inflammation and its levels showing a positive correlation with immune system activation (37).

We used the association of suPAR levels with pruritus intensity, in particular at the follow-up visit, to evaluate its capacity as a prognostic biomarker in patients with CPUO. Higher suPAR levels appear to be associated with higher (in particular average) pruritus intensity in CPUO in the follow-up, indicating less response to therapy. Furthermore, this group also showed a progression of scratch lesions. We are not aware of any previous similar reports. Of course, the linearity implied by the results of the linear regression analysis should be viewed with caution, as a corrected R² of 0.55 might indicate overfitting in this small model. Overall, levels of pruritus intensity in our study were similar to those reported in the literature (38).

Besides being this nonspecific marker of inflammation, suPAR has been associated with several other biological functions, including plasminogen activation, modulation of cell adhesion, migration, and proliferation (39). Hence, it might be conceivable that suPAR could have a specific pathogenetic effect in CPUO, but more research is needed to assess this, surpassing the scope of this study. For now, our results support that CPUO, even after exclusion of e.g. chronic kidney disease, is associated with chronic systemic inflammation as illustrated by suPAR levels.

As discussed above, adverse childhood experiences are a major risk factor of PSS (10–12). One study has also linked adverse childhood experiences to AD (40), while the role of such experiences in CPUO has not been formally described. Interestingly, elevated levels of suPAR were associated with adverse childhood experiences (41, 42) suggesting that suPAR might link this psychological risk factor to CP. We found non-significantly elevated levels of self-reported adverse childhood experiences in CPUO compared to HC (by Adverse Childhood Experiences – German scale, 1.7 ± 1.8 vs. 1.2 ± 1.8). Elevations of suPAR have also been described in other mental disorders such as depression and psychotic disorders (43, 44) and we determined significantly higher levels of depression in CPUO compared to HC (by Public Health Questionnaire 9, 6.4 ± 5.3 vs. 3.3 ± 4.3, *p* = 0.01). Unfortunately, no significant correlations between both risk factors - adverse childhood experiences and depression - and the levels of suPAR were detected (data not shown).

Chronic inflammation is linked to or possibly one driver of biological aging (45). Hence it is not surprising that suPAR as a marker of chronic inflammation appears to increase with age (46). Over our entire sample, there was a trend towards a positive correlation between chronological age and suPAR (*r* = 0.18, *p* = 0.07, data not shown). As the term ‘CPUO’ encompasses ‘pruritus of the elderly’ (4), it is not surprising that our CPUO sample was much older than participants with cAD. As age groups (<65 and ≥65 years) are often used to acknowledge systemic inflammatory changes that comes with aging, we also included this as a covariant in our analysis (45, 47). We also included several other possible biological covariates of suPAR levels. These did either not differ significantly between the groups (e.g. sex ratio, smoking) or did not influence results. It has to be noted however that other possible confounders, such as pro-inflammatory comorbidities and influences of co-medication on suPAR, may exist, which could not be assessed in this analysis. Overall, the impact of possible confounders should be carefully addressed in future studies with larger cohorts. The relatively small sample size of our study, due to the complexity of visits in the overall SOMA.PRU study and the relative scarcity of CPUO in clinical practice, is an important limitation regarding possible confounders and statistical certainty. As this was a secondary, exploratory analysis, no *a priori* power calculation was performed.

Concerning further limitations, patients with CP were only recruited at a single university hospital and only belonged to two phenotypes (CPUO and cAD), hence results may not be transferable to patients in other settings or with other CP types/known etiologies. Despite the longitudinal design of our study with a follow-up after six months and a repeated assessment of pruritus intensity and SSS, blood samples were only drawn at baseline, so the intra-individual course of suPAR levels is not assessed in our study. Additionally, serum levels of suPAR might not fully reflect dermal expression of this biomarker, which might also be important in pruritus.

## 5 Conclusions

In an exploratory, prospective study of patients with pruritus, we found indications of serum levels of suPAR being associated with the subtype of CPUO and its clinical course over six months. To our knowledge, this is the first link of pruritus on non-lesional skin to the biomarker suPAR of systemic chronic inflammation. These findings should be replicated in larger cohorts, focusing on age, possible subtypes of non-lesional CP (neurogenic, cholestasis etc.) and repeated suPAR measurements, to gain further understanding on the value of suPAR as a biomarker in CPUO. Recent advantages in diagnostic methods could enable rapid determination of suPAR (37), increasing the clinical applicability of suPAR and enhancing its value as a potential biomarker especially for CPUO.

## Data Availability

The original contributions presented in the study are included in the article/**Supplementary Material**. Further inquiries can be directed to the corresponding author.

## Supplementary material

Supplementary Table 1 Sociodemographic and clinical data of 78 participants with chronic atopic dermatitis, chronic pruritus of unknown origin and skin-healthy controls at the 6-months follow-up.

Supplementary Table 2 Comparison of 35 patients with chronic pruritus of unknown origin and high versus low serum levels of soluble urokinase plasminogen activator receptor.
