# Supplementary material for: Association of the inflammatory marker suPAR with chronic pruritus of unknown origin – data from the SOMA.PRU study
Source: Front Immunol. 2026 Jun 16;17:1804748. doi: 10.3389/fimmu.2026.1804748 (PMC13314524; doi:10.3389/fimmu.2026.1804748)
Supplement: Supplementary Table 1 — Sociodemographic and clinical data of 78 participants with chronic atopic dermatitis, chronic pruritus of unknown origin and skin-healthy controls at the 6-months follow-up. [file Table1.docx]

**Supp. Table 1**: Sociodemographic and clinical data of 78 participants with chronic atopic dermatitis, chronic pruritus of undetermined origin and skin-healthy controls at the 6-months follow-up.

|  | cAD | | CPUO | | HC | | Significance (cAD vs. CPUO) |
| --- | --- | --- | --- | --- | --- | --- | --- |
|  | *n* | Mean ± SD | *n* | Mean ± SD | *n* | Mean ± SD |  |
| age | 27 | 37.6 ± 19.9 | 28 | 64.0 ± 10.6 | 14 | 41 ± 16.2 | *p* < 0.001 |
| sex | 33 | 20 female,  13 male | 29 | 27 female,  12 male | 16 | 12 female,  4 male | *p* = 1 |
| average pruritus | 27 | 3.8 ± 1.9 | 28 | 4.4 ± 2.7 | 14 | n. a. | *p* = 0.36 |
| worst pruritus | 27 | 5.8 ± 2.3 | 28 | 6.1 ± 3.1 | 14 | n. a. | *p* = 0.66 |
| SSS | 27 | 2.5 ± 1.9 | 28 | 2.3 ± 2.9 | 14 | n. a. | *p* = 0.69 |

6MFU: 6-month follow-up; CPUO: chronic pruritus of undetermined origin; NRS: Numeric Rating Scale; suPAR: soluble urokinase plasminogen activator receptor.
