# Supplementary material for: Association of the inflammatory marker suPAR with chronic pruritus of unknown origin – data from the SOMA.PRU study
Source: Front Immunol. 2026 Jun 16;17:1804748. doi: 10.3389/fimmu.2026.1804748 (PMC13314524; doi:10.3389/fimmu.2026.1804748)
Supplement: Supplementary Table 2 — Comparison of 35 patients with chronic pruritus of unknown origin and high versus low serum levels of soluble urokinase plasminogen activator receptor. [file Table2.docx]

**Supp. Table 2:** Comparison of 35 patients with chronic pruritus of undetermined origin and high versus low serum levels of soluble urokinase plasminogen activator receptor

|  | CPUO high suPAR | | | CPUO low suPAR | | | significance | |
| --- | --- | --- | --- | --- | --- | --- | --- | --- |
|  | *n* | Mean ± SD | | *n* | Mean ± SD | |  |  |
| age | 18 | 68.1 ± 8.5 | | 17 | 59.6 ± 11.1 | | *p* = 0.02 | |
| BMI | 17 | 28.6 ± 3.8 | | 17 | 27.3 ± 6.5 | | *p* = 0.49 | |
| smoking (no \| occasionally \|regularly) | 15 | 13 \| 1 \| 1 | | 15 | 14 \| 0 \| 1 | | *p* = 1 | |
| creatinine | 18 | 0.9 ± 0.17 | | 17 | 0.76 ± 0.14 | | *p* = 0.01 | |
|  |  | Baseline | 6MFU |  | Baseline | 6MFU | high suPAR | low suPAR |
| average pruritus | 18 | 6.3 ± 1.8 | 5.6 ± 2.5 | 17 | 5.7 ± 2.1 | 3.2 ± 2.4 | *p* = 0.017 | *p* = 0.3 |
| worst pruritus | 18 | 7.6 ± 1.9 | 7.3 ± 2.4 | 17 | 6.8 ± 2.0 | 4.9 ± 3.4 | *p* = 0.043 | *p* = 0.2 |
| SSS | 18 | 1.2 ± 1.5 | 3.2 ± 3.5 | 17 | 1.1 ± 2.0 | 1.3 ± 1.9 | *p* = 0.09 | *p* = 0.9 |

6MFU: 6-months follow-up; BMI: Body Mass Index; SSS: Scratch Sign Score; suPAR: soluble urokinase plasminogen activator receptor
